# Physiological changes and growth behavior of *Corynebacterium glutamicum* cells in biofilm

**DOI:** 10.3389/fmicb.2022.983545

**Published:** 2022-08-30

**Authors:** Di Zhang, Jiawen Shen, Xiwei Peng, Shansong Gao, Zhenyu Wang, Huifang Zhang, Wenjun Sun, Huanqing Niu, Hanjie Ying, Chenjie Zhu, Yong Chen, Dong Liu

**Affiliations:** ^1^State Key Laboratory of Materials-Oriented Chemical Engineering, College of Biotechnology and Pharmaceutical Engineering, Nanjing Tech University, Nanjing, China; ^2^Jiangsu National Synergetic Innovation Center for Advanced Materials (SICAM), Nanjing Tech University, Nanjing, China

**Keywords:** *Corynebacterium glutamicum*, biofilm cells, cell division, cell size, longterm fermentation

## Abstract

Biofilm cells are well-known for their increased survival and metabolic capabilities and have been increasingly implemented in industrial and biotechnological processes. *Corynebacterium glutamicum* is one of the most widely used microorganisms in the fermentation industry. However, *C. glutamicum* biofilm has been rarely reported and little is known about its cellular basis. Here, the physiological changes and characteristics of *C. glutamicum* biofilm cells during long-term fermentation were studied for the first time. Results showed that the biofilm cells maintained stable metabolic activity and cell size was enlarged after repeated-batch of fermentation. Cell division was slowed, and chromosome content and cell proliferation efficiency were reduced during long-term fermentation. Compared to free cells, more biofilm cells were stained by the apoptosis indicator dyes Annexin V-FITC and propidium iodide (PI). Overall, these results suggested slow-growing, long-lived cells of *C. glutamicum* biofilm during fermentation, which could have important industrial implications. This study presents first insights into the physiological changes and growth behavior of *C. glutamicum* biofilm cell population, which would be valuable for understanding and developing biofilm-based processes.

## Introduction

Biofilm is microbial communities that are enclosed in self-produced extracellular polymer substances (EPS) and attached to surfaces (Costerton et al., 1995). Biofilm has good water-holding capacity and permeability, which can stabilize the hydration layer on the cell surface and create favorable growth microenvironment for cells (Harrison et al., 2007; Flemming and Wingender, 2010; Flemming et al., 2016). Thus, biofilm can be considered as a living system with the best biocompatibility. They can be formed in natural and medical environments with harmful effects (López et al., 2010). On the other hand, microorganisms in the form of biofilm can be very advantageous in industrial fermentation. For example, Chen et al. (2019) enhanced the biofilm formation of engineered *Escherichia coli* by overexpressing the *fimH* gene and the yield of L-threonine was improved from 10.5 to 17.5 g/L during continuous fermentation. Wang et al. (2010) used the biofilm form of *Rhizopus oryzae* for fermentation. Compared with free-cell (FC) fermentation, lactic acid production by the *R. oryzae* biofilm was increased by 70% and production time was shortened by 33%. Chen et al. (2013) reported that the ethanol productivity achieved by the *Saccharomyces cerevisiae* biofilm during repeated-batch fermentation in packed-bed reactors was 3.6-fold higher than that of free cells, and that the biofilm cells showed enhanced production stability.

Cells in biofilm can enhance the cooperation for growth, metabolism, and physiological behavior through quorum sensing and other mechanisms, improving the survival and metabolic ability of the whole population (Flemming and Wingender, 2010; Flemming et al., 2016). Biofilm cells exhibited distinct characteristics compared with planktonic cells in gene expression, cell proliferation, morphology development and metabolism. Liang et al. (2020) studied the *S. cerevisiae* biofilm during fermentation process and found that biofilm cells displayed an altered growth cycle, wherein most of the biofilm cells adhering to carrier surfaces were in the G2/M phase. In *Clostridium acetobutylicum* biofilm, Liu et al. (2018) found that cells in biofilm during continuous cultivation underwent significant morphological changes: from short rods to long chains. Uppuluri et al. (2010) examined the morphology of *Candida albicans* cells dispersed from biofilms in RPMI, YNB, and YPD medium and found RPMI-grown biofilms were normally more “filamentous”.

Although biofilm formation and physiological characteristics of biofilm cells have been extensively studied in many industrial strains like *E. coli*, *S. cerevisiae*, and *B. subtilis*, these are less studied in *Corynebacterium glutamicum*. *C. glutamicum* is one of the most widely used strains for the industrial production of various amino acids and other chemicals. So far, there are few reports on the biofilm of *C. glutamicum*. Recently we engineered *C. glutamicum* with inactivated extracellular nuclease gene, which promoted biofilm formation and increased the production of L-proline by 60% (Ren et al., 2020). Ha et al. (2017) reported that the biofilm formation ability of *C. glutamicum* ATCC13032 was increased 4–5 times by inactivating the gene *NCg12909*. This increased the cell mass while the production of L-lysine was not reduced. Despite the potential benefits provided by the biofilm in industrial processes, the physiological adaptation of *C. glutamicum* biofilm cells has not been characterized yet. How they change in terms of growth, morphology, metabolism, replication, etc. remains to be elucidated.

In this study, the profiles such as cell morphology, division, proliferation and deoxyribonucleic acid (DNA) content of *C. glutamicum* biofilm cells during long-term repeated-batch fermentation were investigated, to reveal the dynamics of cell population and cellular basis underlying fermentation process. This study provides novel insights for understanding the physiological properties of *C. glutamicum* biofilm cells and would be valuable for optimization of the biofilm fermentation system and its industrial application.

## Materials and methods

### Strain, culture medium and growth conditions

The plasmids and strains used in this study are listed in Table 1. Both a wild-type model strain *C. glutamicum* ATCC 13032 and an L-lysine producing industrial strain *C. glutamicum* 0206 (Cg-0206) were studied in this research. *C. glutamicum* ATCC 13032 was used to construct recombinant *C. glutamicum* ATCC 13032-(FtsZ-EGFP) to trace the expression of the gene *ftsZ*. Its culture medium was: glucose 10 g/L, corn pulp 20 g/L, KH_2_PO_4_ 1.2 g/L, (NH_4_)_2_SO_4_ 30 g/L, MgSO_4_ 7H_2_O 0.4 g/L, Urea 2 g/L. The industrial Cg-0206 strain used a production medium: glucose 80 g/L, yeast extract 8 g/L, K_2_HPO_4_⋅3H_2_O 1.5 g/L, (NH_4_)_2_SO_4_ 13.2 g/L, MgSO_4_ 7H_2_O 0.6 g/L, FeSO_4_⋅7H_2_O 0.1g/L, urea 15 g/L, MOPS 42 g/L, copper sulfate 0.9 ml/L, zinc sulfate 1 mg/L, biotin 1.8 mg/L, vitamin B_1_ 9 mg/L, manganese sulfate 150 g/L, calcium d-pantothenate 9 mg/L, niacinamide 60 mg/L. All media were sterilized at 115°C for 20 min.

**TABLE 1:** Strains and plasmids used in this study.

Strains or plasmids	Relevant characteristics	Reference/sources
**Strains**		
*C. glutamicum* ATCC13032	Wild-type strain	Laboratory stock
*C. glutamicum* ATCC13032-(FtsZ-EGFP)	*C. glutamicum* ATCC13032 with EGFP-tagged FtsZ	This study
*C. glutamicum* CICC 0206	L-lysine producing strain	Lei et al., 2021
*E. coli* DH5α	Plasmids holding strain	Laboratory stock
**Plasmids**		
pK18mobsacB	Integration vector, ori pUC, Km^r^, mob sacB	Schäfer et al., 1994
pK18mobsacB-*egfp*	Integration vector, ori pUC, Km^r^, mob sacB, *egfp*	This study
pUC57-*egfp*	Expression vector, Amp, *egfp*	This study

The plasmid pK18mobsacB was presented by Professor Sheng Yang of Shanghai Academy of Life Sciences, Chinese Academy of Sciences. The pUC57-*egfp* recombinant plasmid was synthesized by Genewiz Biotech Co. (Suzhou, China). All plasmids were introduced into *E. coli* DH5α for cloning. It was grown in Luria-Bertani broth (LB) and incubated at 37°C. *C. glutamicum* strains were grown in LBG medium (LB supplemented with 10 g/L glucose) at 30°C. Antibiotics were added to recombinant strains when necessary: ampicillin 100 μg/mL, kanamycin 15 μg/mL. All other chemicals were of analytical grade and purchased from local suppliers.

### Construction of FtsZ-EGFP expression strain

The gene sequence of EGFP Mgfp5 (Siemering et al., 1996) was codon-optimized for *C. glutamicum* and synthesized on pUC57 plasmid by Genewiz Biotech Co. (Suzhou, China). Chromosomal DNA isolated from *C. glutamicum* ATCC13032 was used as PCR template. PCR primers for this study are listed in Table 2. The preparation of competent cells and electroporation of *C. glutamicum* were performed according to the published methods (Van der Rest et al., 1999). Insertion of *egfp* after the *ftsZ* gene on *C. glutamicum* genome was performed using the non-replicable plasmid pK18mobsacB (Schäfer et al., 1994). For construction of pK18mobsacB-*egfp*, two DNA fragments homologous to *ftsZ* gene fragment and its downstream region were amplified using the primer pairs: R-arm-F/R-arm-R (for the *ftsZ* gene fragment) and L-arm-F/L-arm-R (for *ftsZ* downstream fragment), respectively. The *egfp* DNA was amplified from the pUC57-*egfp* using the primer pairs: *egfp*-F/*egfp*-R. Then the three fragments were assembled by overlap PCR. The resulting product was ligated to *HindIII/EcoRI*-linearized plasmid pK18mobsacB based on homologous recombination using a ClonExpressTMI One Step Cloning Kit (Vazyme Biotech Co., Nanjing, China). The resulting pK18mobsacB-*egfp* was verified by sequencing. The pK18mobsacB-*egfp* was transferred to *C. glutamicum* by electroporation as described previously (Eggeling and Bott, 2005). Integration of pK18mobsacB-*egfp* into the chromosome was confirmed by selection on LB plates containing kanamycin. Kanamycin-resistant colonies were grown overnight in liquid LB and spread on LB plates containing 10% (w/v) sucrose. Finally, kanamycin-sensitive and sucrose-resistant colonies were selected and the double crossover events were confirmed by PCR (Moon et al., 2005), using the primer pair ATCC13032-(FtsZ-EGFP)-F/ATCC13032-(FtsZ-EGFP)-R. The *C. glutamicum* ATCC 13032-(FtsZ-EGFP) was observed under an inverted fluorescence microscope MF53 (MSHOT, China). A CytoFLEX flow cytometer (FCM) (Beckman Coulter, America) was also used to analyze the FtsZ-EGFP fluorescence at the single cell level.

**TABLE 2:** Oligonucleotides used in this study^a^.

Oligos	Sequence(5′→3′)
R-arm-F	ACGACGGCCAGTGCCAAGCTTCAGGCAGA AGAAGGCATC
R-arm-R	TTCACCCTTGGACATACTACCTCCGCCCCC CTGGAGGAAGCTGGGTACAT
*egfp*-F	CCCAGCTTCCTCCAGGGGGGCGGAGGTAG TATGTCCAAGGGTGAAG
*egfp*-R	TCTCCTTCTTAATTATTACTTGTACAGTTCA TCC
L-arm-F	GGATGAACTGTACAAGTAATAATTAAGAAG GAGAATAGACTTATCC
L-arm-R	CTATGACATGATTACGAATTCACGGGGTGG AATTTG
ATCC13032-(FtsZ-EGFP)-F	CACTGACCATTGGTGTTGTGAC
ATCC13032-(FtsZ-EGFP)-R	TGCGGTGACACCAAACTCTTG

^a^Restriction sites: AAGCTT, HindIII; GAATTC, EcoRI.

### Fermentation method

FC fermentation was performed in 500-mL shaking flasks containing 50 mL of working volume at 30°C and 200 rpm, with 10% (v/v) inoculum. For free cell repeated-batch fermentation, half of the fermentation broth at the end of a batch of fermentation was replaced with fresh medium to start the next batch of fermentation. Generally, the fermentation broth was replaced with fresh fermentation broth every 24 h.

Biofilm-cell (BC) fermentation was performed under the same conditions for free cell fermentation, except that 40 pieces of cotton fiber were added into each flask as biofilm carriers. The purchased cotton fiber (Sunvim Co., Shandong, China) was washed with pure water, dried in a 65°C constant temperature oven. It was cut into pieces with a size of 1.1 cm × 1.1 cm, approximately 0.05 g per piece. For BC repeated-batch fermentation, the fermentation broth was poured out at the end of fermentation while the biofilm carriers were retained. Then, fresh medium was added to start the next batch of fermentation. Generally, the fermentation broth was replaced with fresh fermentation broth every 24 h. When necessary, the cotton fiber carriers were collected and rinsed with PBS (pH = 7.4) to observe biofilm cell growth and division.

### Analysis of product and cell concentrations

The initial glucose concentration for Cg-0206 fermentation was 80 g/L. The concentrations of L-lysine and residual glucose were detected by an SBA-40E biosensor analyzer (Shandong, China). The total cell concentration in biofilm fermentation broth was calculated as the sum of the carrier-attached (biofilm) cell concentration and the FC concentration in the bulk liquid. To determine the carrier-attached cell concentration, each biofilm carrier (each piece of cotton towel was about 0.05 g of dry mass, 0.3 mL of wet volume) was violently rinsed in 5 mL of pure water for three times, and then the OD_600_ value of the 15 mL eluent was measured and an equivalent OD_600_ value was obtained.

### Analysis of cell size

Relative cell size was analyzed using the FCM forward scattered light channel, whose value reflects the cell size (Müller and Nebe-von-Caron, 2010). That was, the larger the cell volume, the greater the FSC value. In this study, the cell size was expressed as the mean value of forwarding scattering (FSC) histogram.

### Analysis of cell proliferation efficiency

Dilution of carboxyfluorescein diacetate succinimidyl ester (CFDA-SE) fluorescence inside cells was used for investigating cell division rate. The staining procedures for *C. glutamicum* cells at the log phase were modified from the procedures described in the CFDA-SE kit (KeyGEN BioTECH, China). For free cells, 500 μL of fermentation broth was taken directly, enriched by centrifugation and incubated for 30 min at 37°C in 2 mL of CFDA-SE cell labeling solution and 2 mL of CFDA-SE storage solution supplied by the kit. After incubation, cells were harvested and resuspended in fresh fermentation broth for growth. For biofilm cells, a piece of cotton towel attached with biofilm cells was taken from the log phase fermentation broth and put into PBS (pH = 7.4), rubbed and washed, and the cells were rinsed as thoroughly as possible. Cells were harvested by centrifugation at 4,000 rpm for 10 min and incubated for 30 min at 37°C in 2 mL of CFDA-SE cell labeling solution and 2 mL of CFDA-SE storage solution supplied by the kit. After incubation, cells were harvested and resuspended in fresh fermentation broth, including a cotton towel for cell adhesion. Subsequently, cells were analyzed at predetermined time intervals by FCM through the FL1 channel for green fluorescence with excitation wavelength of 488 nm and emission wavelength of 518 nm.

### Analysis of deoxyribonucleic acid content

The content of DNA was determined by the fluorescent dye 2-(4-Amidinophenyl)-6-indolecarbamidine dihydrochloride (DAPI) (Beyotime Biotechnology, China), according to the manufacturer’s instructions. After staining, cells were collected by centrifugation and washed twice with PBS (pH = 7.4). Then, the PBS-resuspended cells were immediately observed by FCM Fluorescence 6 (FL6) detection channel.

### Live-dead staining

Annexin V-FITC/PI apoptosis kit (Boster Biological Technology Co., Ltd.) was used for assay of apoptosis-like death. Cell samples of different fermentation periods were diluted to an OD_600_ of 0.25 for staining (Choi et al., 2016), according to the instructions of the kit. The FCM fluorescence 1 (FL1) detection channel and fluorescence 2 (FL2) detection channel were used to detect Annexin V-FITC and propidium iodide (PI) fluorescence, respectively.

## Results

### Sustainable metabolic activity of *Corynebacterium glutamicum* biofilm cells during repeated-batch fermentation

**FIGURE 1:**
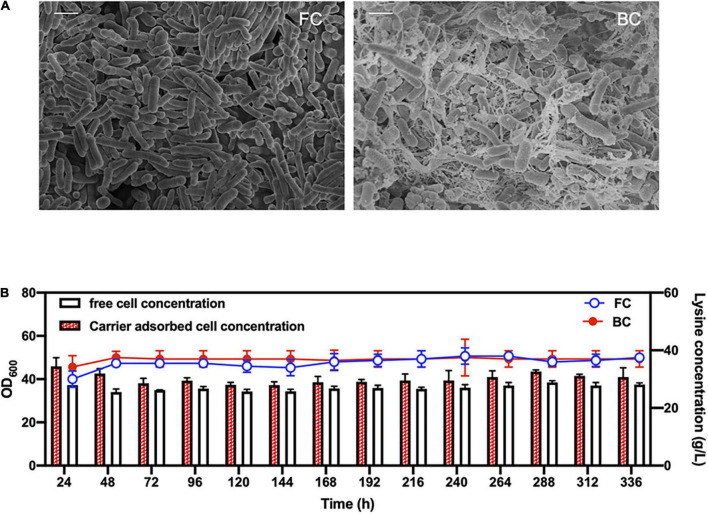
Performance of *C. glutamicum* biofilm cells in fermentation. **(A)** SEM images of free cells (FC) and carrier-attached biofilm cells (BC) during fermentation. Scale bar, 1 μm. **(B)** Lysine production of Cg-0206 cells and cell concentrations of Cg-0206 biofilm cells during repeated-batch fermentation.

Biofilm cells were well-immobilized by EPS on carrier surfaces (Figure 1A). So, compared to traditional fermentation by free cells, fermentation by biofilm cells could be operated continuously or in a repeated batch fermentation mode. Fermentation performances of L-lysine producing Cg-0206 biofilm cells in 14 repeated batches were shown in Figure 1B. Glucose could be completely consumed in 24 h and the average lysine production was maintained around 37 g/L in each batch (Figure 1B), which was equivalent to free cell production (Figure 1B), suggesting a sustainable metabolic activity of the biofilm cells. The cell concentration (OD_600_) in the bulk liquid was around 35 (Figure 1B). However, the cell concentration in biofilm on the carrier was generally higher than in the bulk liquid. With repeated-batch fermentation, the cell concentration in biofilm gradually stabilized, with a final OD_600_ of around 40. Therefore, metabolic activity and growth of biofilm cells remained stable during repeated-batch fermentation. On this basis, the physiological characteristics and morphology of biofilm cells in long-term repeated-batch fermentation were studied.

### Enlarged cell size of biofilm cells during long-term fermentation

The change in size of Cg-0206 biofilm cells as well as free cells throughout a single batch fermentation over 133 h was first studied, using a CytoFLEX FCM whose FSC value is proportional to cell size. Results showed that the average size of biofilm cells was 32–88% larger than that of free cells at different time points during the early growth phase (12–18 h) (Supplementary Figure 1). However, this difference was apparently diminished after the cells entered stationary phase. After entering the stationary phase (22–133 h), the size of biofilm cells was significantly reduced and comparable to that of free cells, which was consistent with a theory that cells become smaller in the stationary phase (Neumeyer et al., 2013).

**FIGURE 2:**
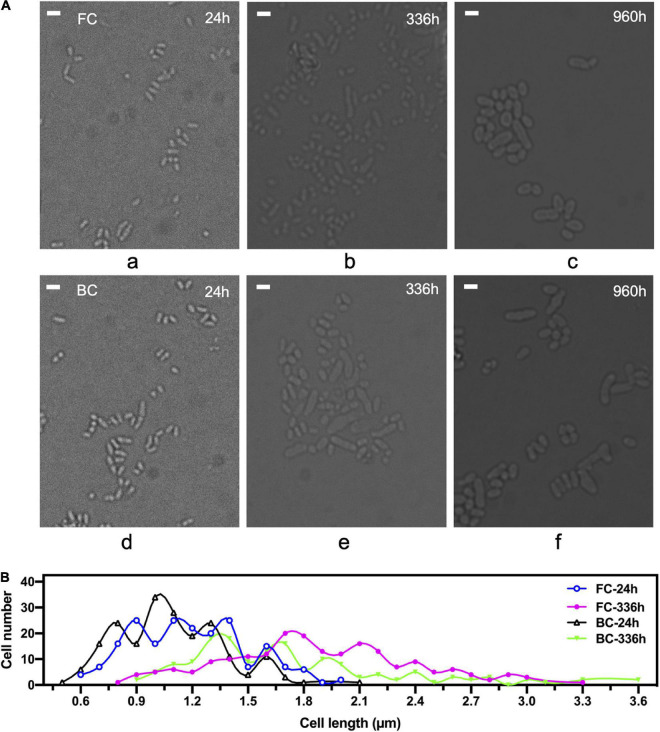
Morphological changes of *C. glutamicum* cells over time during long-term repeated-batch fermentation. FC, free cells; BC, biofilm cells. **(A)** Observation of cells under a microscope. Scale bar, 2 μm. **(B)** Statistical analysis of cell length under the microscope. 200 cells were counted and the cell length was measured by Image J software. The times indicate sampling time points at which cells of exponential growth phase during a repeated batch were taken.

Next, morphological changes of biofilm cells throughout long-term repeated batch fermentation over 960 h was investigated. Results showed that during the long-term repeated batch fermentation, the biofilm cells were enlarged over time (Figure 2A). The average length of biofilm cells at 24 h was around 1.6 μm, whereas it was increased to 2.6 μm at 960 h (Figure 2A). The elongation of biofilm cells was mainly due to prolonged fermentation time rather than effects from carrier, because it was shown that free cells also grew larger after long-term repeated batch fermentation. In general, the morphology of biofilm cells after long-term fermentation did not differ greatly from that of free cells undergoing long-term fermentation as well. However, some exceptionally large cells appeared relatively early during the biofilm fermentation, reaching a length as long as 3.6 μm at 336 h (Figure 2B), which was less evident in free cell fermentation. Morphological heterogeneity of both biofilm and free cells after long-term fermentation was observed, but the heterogeneity in the cell shape of biofilm cells was generally more apparent.

### Slowed cell division within biofilm

**FIGURE 3:**
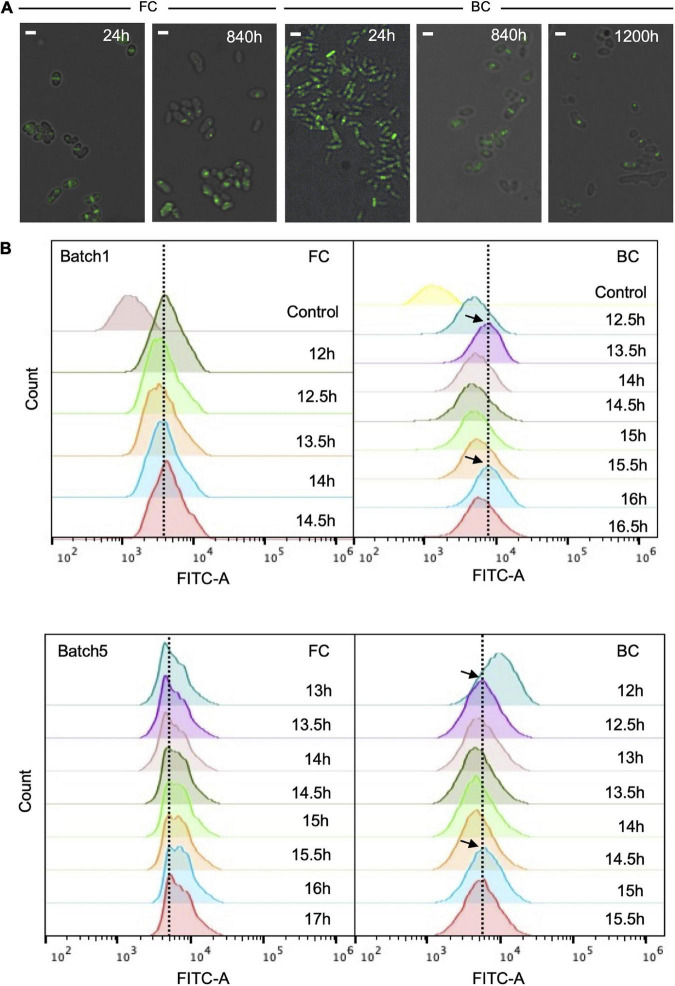
Fluorescence of *C. glutamicum* ATCC13032- (FtsZ-EGFP) cells during long-term repeated-batch fermentation. **(A)** The fluorescence pictures overlapped with bright field pictures. BC, biofilm cells; FC, free cells. The fluorescent septa or foci indicate the formation of Z-rings at different time points. Scale bar, 2 μm. **(B)** Fluorescence intensity histograms of flow cytometry analysis. Cells were analyzed at the exponential growth phase (12–17 h) of each batch. The black arrows indicate possible oscillations in fluorescence intensity of biofilm cells. Fluorescence intensity of wild *C. glutamicum* ATCC13032 without EGFP fusion was used as control.

Cell division of biofilm cells during long-term repeated-batch fermentation was studied by monitoring the cell division protein FtsZ fluorescently tagged with enhanced green fluorescent protein (EGFP). To observe the cells by fluorescence microscopy, the native *ftsZ* gene on *C. glutamicum* ATCC13032 genome was tagged with an EGFP gene. The growth of *C. glutamicum* ATCC13032-(FtsZ-EGFP) was comparable to that of the original strain (Supplementary Figure 2), indicating that fusion of EGFP with FtsZ protein did not disturb cell growth and the FtsZ-EGFP fusion was functional. Results showed that at the early culture stage (e.g., 24 h), the fluorescence intensity of cells was high, and there was a clear septum at the middle of most cells (Figure 3A). Fluorescent cells accounted for more than 85% of the total cell number. This indicated that most of cells were undergoing division and the period of cell division was short. After the long-term fermentation (e.g., 840 h), the fluorescent population of the biofilm cells was apparently reduced, but still accounted for 30–50% of the total cell number. This ratio was roughly stable throughout a fermentation period of at least 1,200 h (Figure 3A). This indicated that the cells immobilized within biofilm could well maintain cell division capacity throughout the long-term fermentation, which was important for sustainable productivities. On the other hand, the ratio of dividing cells in biofilm after long-term fermentation was lower than during early-stage fermentation, and was also lower than that of FC fermentation. Furthermore, a fluorescent focus instead of a septum was often observed at the middle of cells undergoing long-term fermentation, indicating slower FtsZ-ring (Z-ring) assembly. Altogether, these results showed that cell division in biofilm was slowed down during long-term fermentation. This was probably because mature biofilm usually contained a large proportion of long-lived cells, thus cell division was slowed down to reduced cell renewal.

The cells with EGFP-tagged FtsZ grown in biofilm were also subjected to flow cytometry to detect their fluorescence. To do so, samples were taken 12 h after the start of each batch, consecutively at 30 min intervals. Interestingly, oscillations in fluorescence intensity were observed with a period of roughly 2.5 h (Figure 3B). By contrast, no apparent oscillations in FC culture were observed. These oscillations possibly indicated that coordinated cell division occurred within the biofilm. Since the period of cell division was difficult to detect for biofilm system, the period of oscillation in divisome may be used to tentatively represent the period of cell division. This period was longer than the generation time (2.0 h) of free cells that was calculated from the growth curve during batch fermentation (Supplementary Figure 2). Again, this indicated that the cell division of biofilm cells was slower than that of free cells.

### Reduced chromosome content in biofilm cells

**FIGURE 4:**
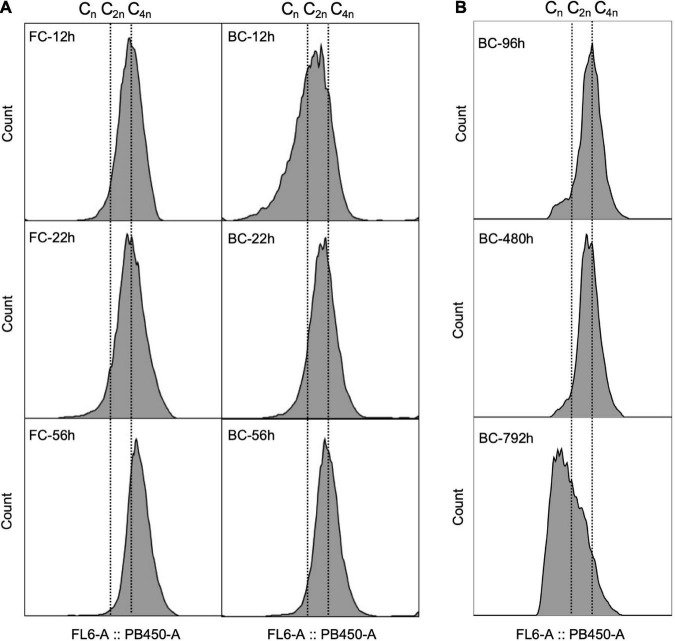
Comparison of DNA patterns between *C. glutamicum* biofilm cells (BC) and free cells (FC) during fermentation. **(A)** DNA patterns of cells taken at different time points during a single-batch fermentation. The chromosome equivalents were determined according to Neumeyer et al. (2013) and expressed as C_*n*_, wherein represents the number of chromosome equivalents. **(B)** DNA patterns of *C. glutamicum* biofilm cells taken at time points of log phase during repeated-batch fermentation.

Chromosome replication is tightly regulated with cell division. *C. glutamicum* was recently recognized as a diploid strain with two pole-attached chromosomes (C_2n_). Furthermore, *C. glutamicum* was shown to overlap chromosome replication periods during fast growth, a phenomenon termed multifork replication (Neumeyer et al., 2013; Böhm et al., 2017), giving the cells multiple chromosome equivalents. Here, the DNA of *C. glutamicum* biofilm cells was stained with DAPI and chromosome equivalents were analyzed as described previously (Neumeyer et al., 2013). Results showed that during the early culture stage, around 20–45% of the biofilm cells contained more than four chromosome equivalents, indicating a proportion of fast-growing cells existed in biofilm. However, this proportion was relatively smaller compared to that of free cells (Figure 4A). During the long-term fermentation, the proportion of biofilm cells with more than four chromosome equivalents diminished (Figure 4B), indicating a reduced growth rate over time. A similar DNA pattern was observed in the industrial strain Cg-0206 throughout long-term fermentation (Supplementary Figure 3). In addition, compared to free cells, the peaks for biofilm cells were more irregular with broader width, indicating biofilm cells were more heterogeneous in chromosome content.

### Cell proliferation efficiency in repeated-batch fermentation

**FIGURE 5:**
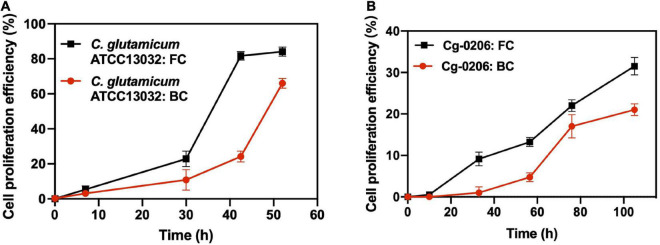
Comparison of cell proliferation efficiency between biofilm cells (BC) and free cells (FC). **(A)** *C. glutamicum* ATCC13032 cells taken at the log phase of batch 2 (59 h). **(B)** *C. glutamicum* 0206 cells taken at the log phase of batch 1 (16 h). Cells were collected and stained with CFDA-SE, and then resuspended into fresh culture medium (0 h) for proliferation. Dilution of fluorescence was recorded at predetermined time intervals using FCM. Cell proliferation efficiency was defined as the percentage of cells whose fluorescence intensity fell into the control region (Supplementary Figure 4).

In order to study the proliferation efficiency of biofilm cells during long-term fermentation, biofilm cells from different batches were labeled with a stable fluorescent dye CFDA-SE. Then the dilution of the dye over time due to cell proliferation was monitored by the FCM (Supplementary Figure 4). Results showed that for both *C. glutamicum* ATCC13032 and Cg-0206 strains, the proliferation efficiency of biofilm cells was lower than that of free cells (Figure 5). At the same time, it was observed that although the proliferation efficiency of detached biofilm cells was lower than that of free cells, they still displayed active proliferation after prolonged culture. Also, the fluorescence was evenly distributed and diluted during cell proliferation (Supplementary Figure 4). These results indicated that although the cells within biofilm were kept in a slow-growing state, most of them retained the ability to divide.

### Altered Annexin V Fluorescein isothiocyanate and propidium iodide staining pattern of biofilm cells

**FIGURE 6:**
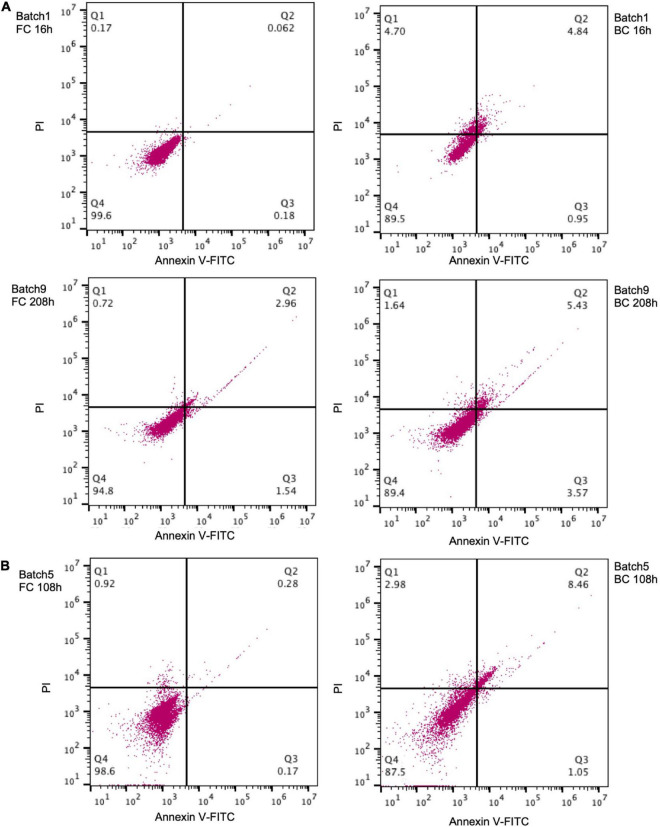
Annexin V-FITC and PI staining of *C. glutamicum* biofilm cells (BC) and free cells (FC). **(A)** *C. glutamicum* ATCC13032 cells taken from batch 1 (16 h) and batch 9 (208 h) of fermentation. **(B)** Cg-0206 cells taken from batch 5 (108 h) of fermentation. The cell population in the Q4 region indicates negative cells, the Q1 region indicates PI positive cells, the Q2 region indicates Annexin V-FITC and PI positive cells, and the Q3 region indicates Annexin V-FITC positive cells. The number indicates percent of cells in each region.

PI is a fluorescent dye that can only penetrate damaged cell membranes. Thus PI-positive cells are usually considered “dead.” Annexin V binds to phosphatidylserine that is exposed on the outer membrane of early apoptotic cells. It was also used to detect apoptosis-like death in some bacteria like *E. col*i (Choi et al., 2016). *C. glutamicum* could be selectively stained by the FITC-labeled Annexin V (Supplementary Figure 5). During the early fermentation stage (e.g., 16 h), the proportion of PI-positive (9.5%) and Annexin V-positive (5.7%) biofilm cells was much greater than the proportion of PI-positive (0.23%) and Annexin V-positive (0.24%) free cells (Figure 6A). After prolonged fermentation (e.g., 208 h), the proportion of PI-positive (7.1%) and Annexin V-positive (9.0%) biofilm cells was relatively stable, but was still greater than the proportion of PI-positive (3.6%) and Annexin V-positive (4.5%) free cells. Similarly, a greater proportion of PI-positive and Annexin V-positive biofilm cells was also observed for another strain Cg-0206 (Figure 6B). The greater proportion of PI- and Annexin V-positive biofilm cells possibly suggested greater proportion of aged or dead cells in biofilm but could also be a result attributed to corynebacterial cell wall composition (discussed below).

## Discussion

The potential of *C. glutamicum* biofilm cells in industrial fermentation has been proven (Ha et al., 2017; Ren et al., 2020). Previously, we deleted *exeM* and *exeM1* in a L-proline-producing strain which promoted biofilm formation (Ren et al., 2020). We also constructed an *exeR* (the homolog of *exeM* and *exeM1*) deletion mutant in an L-lysine-producing strain Cg-0206 (Lei et al., 2021), but it had litter effects on biofilm formation. Here, both the wild-type model strain *C. glutamicum* ATCC13032 and the industrial strain Cg-0206 were studied. The physiological characteristics and growth behavior of the biofilm cells were explained. The size of biofilm cells became larger during long-term fermentation. Morphological changes were commonly observed for various biofilm cells (Kubota et al., 2008). The enlarged cell size of *C. glutamicum* biofilm cells was mainly due to prolonged fermentation time. Indeed, a trend toward larger cells was also observed for *E. coli* cells in a long-term evolution experiment, and larger cells are suggested to be beneficial and important targets of selection (Grant et al., 2021). Enlarged cell size would reduce the surface area-to-volume ratio (SA/V). While a reduced SA/V may lead to decreased uptake of nutrients and export of harmful end products or byproducts, it could on the other hand increase cell tolerance to metabolite stress, since the cell surface and membrane are the major targets of toxic metabolites. From this perspective, the enlarged *C. glutamicum* biofilm cells were beneficial for industrial fermentation.

Slower Z-ring assembly as well as reduced chromosome content and proliferation efficiency all suggested that the growth rate of the biofilm cells was lower than that of the free cells in the process of long-term repeated-batch fermentation. Different from FC system, mature biofilm maintained high cell densities throughout the fermentation process and local nutrients could be limited. Thus, it was reasonable that cell growth in biofilm became slower. Slow-growing cells are less susceptible to stressors and have been demonstrated to increase cell tolerance (Nandy, 2021). More importantly, slow growth typically correlated with slower death (Patra and Klumpp, 2013; Biselli et al., 2020). So, the slow-growing cells in biofilm were thus expected to be long-lived and persist over long time periods of fermentation. Obviously, this would give a decreased cell turnover rate and thus more substrates could be directed to products instead of biomass. This could explain why biofilm-based fermentation apparently increased the amino acid yield (Chen et al., 2019; Ren et al., 2020).

In studying apoptosis-like death, we found that the proportion of PI- and Annexin V-positive cells in biofilm was greater than that for free cells, which may suggest that the apoptosis-like death rate of biofilm was higher. Apoptosis generally serves as a protective mechanism in multicellular systems by removing abnormal cells, thereby preventing deleterious effects that may threaten the survival of the whole population and making the nutrients more available to healthier members of the community (Choi et al., 2016). So, the overall fitness of the biofilm cells could be increased through apoptosis-like death. On the other hand, the greater proportion of PI- and Annexin V-positive cells in biofilm might also be due to the retention of dead cells by biofilm matrix. If this was the case, more elaborate experiments are needed to investigate and optimize its effect on fermentation process. Finally, the PI and Annexin V staining pattern of biofilm cells could also be caused by the specific corynebacterial cell wall structure. Previously, it was observed in *C. glutamicum* that a greater proportion of log phase cells were stained by PI, which was unusual and supposed to be due to loosen cell wall structures during fast growth and cell division (Neumeyer et al., 2013). Therefore, it was also possible that cell wall composition and structures of *C. glutamicum* biofilm cells were altered and more permeable.

## Data availability statement

The original contributions presented in this study are included in the article/Supplementary material, further inquiries can be directed to the corresponding author/s.

## Author contributions

DZ conceived and designed the experiments, performed the laboratory work, analyzed and interpreted the data, and drafted the manuscript. JS, XP, and SG constructed the plasmids and strains, participated in the fermentation experiments, performed the shooting of electron microscope, analyzed the metabolic products, and performed the statistical analysis. ZW, HZ, and WS performed the physiological changes analysis of different time points. HN, HY, CZ, and YC critically revised the manuscript. DL contributed to the experimental design, data interpretation, and critically revised the manuscript. All authors read and approved the final manuscript.
