# Supplementary material for: Physiological changes and growth behavior of Corynebacterium glutamicum cells in biofilm
Source: Front Microbiol. 2022 Aug 30;13:983545. doi: 10.3389/fmicb.2022.983545 (PMC9468548; doi:10.3389/fmicb.2022.983545)
Supplement: Supplementary file 1 [file Table_1.DOCX]

**Supporting Information**

**Fig. S1**
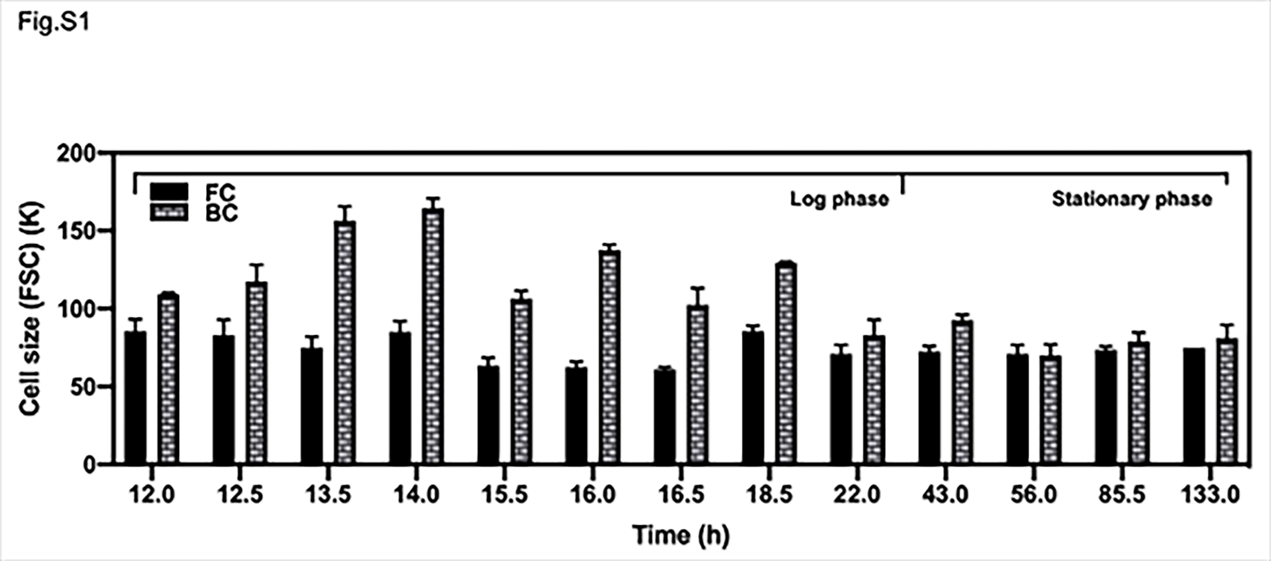


**Fig. S1** The cell size of Cg-0206 was analyzed in a single batch fermentation over 133 h. Relative cell size was analyzed using the CytoFLEX flow cytometer (Beckman Coulter, America) forwards scattering, which reflects the cell size. The cell size was expressed as the mean value of FSC histogram. FC, Free cells; BC, Biofilm cells.

**Fig. S2**


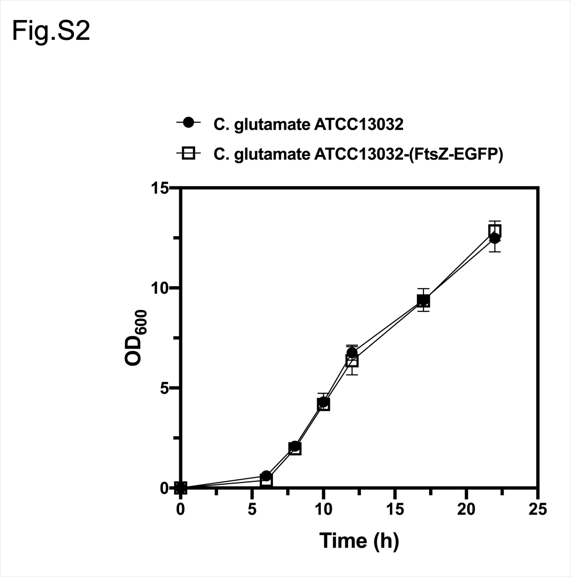


**Fig. S2** *C. glutamate* ATCC 13032 cell concentration. The cell concentration of *C. glutamicum* was determined by an ultraviolet spectrophotometer at 600 nm. Generation time (GT) was calculated by measurement of optical density at 600 nm (OD_600_). The GT value was calculated from the following equation: GT = ln 2/[ln (OD_T2_/OD_T1_)/(*T2* − *T1*)], where *T1* and *T2* were two time points for measurementing OD_T2_ and OD_T1_(Łos et al., 2008). Here, the OD_T2_ value of *C. glutamicum* at 10 h was 4.61 and the OD_T1_ value at 8 h was 2.175. Therefore, GT ≈ 1.845 was finally calculated according to the above equation.

**Fig. S3**

**
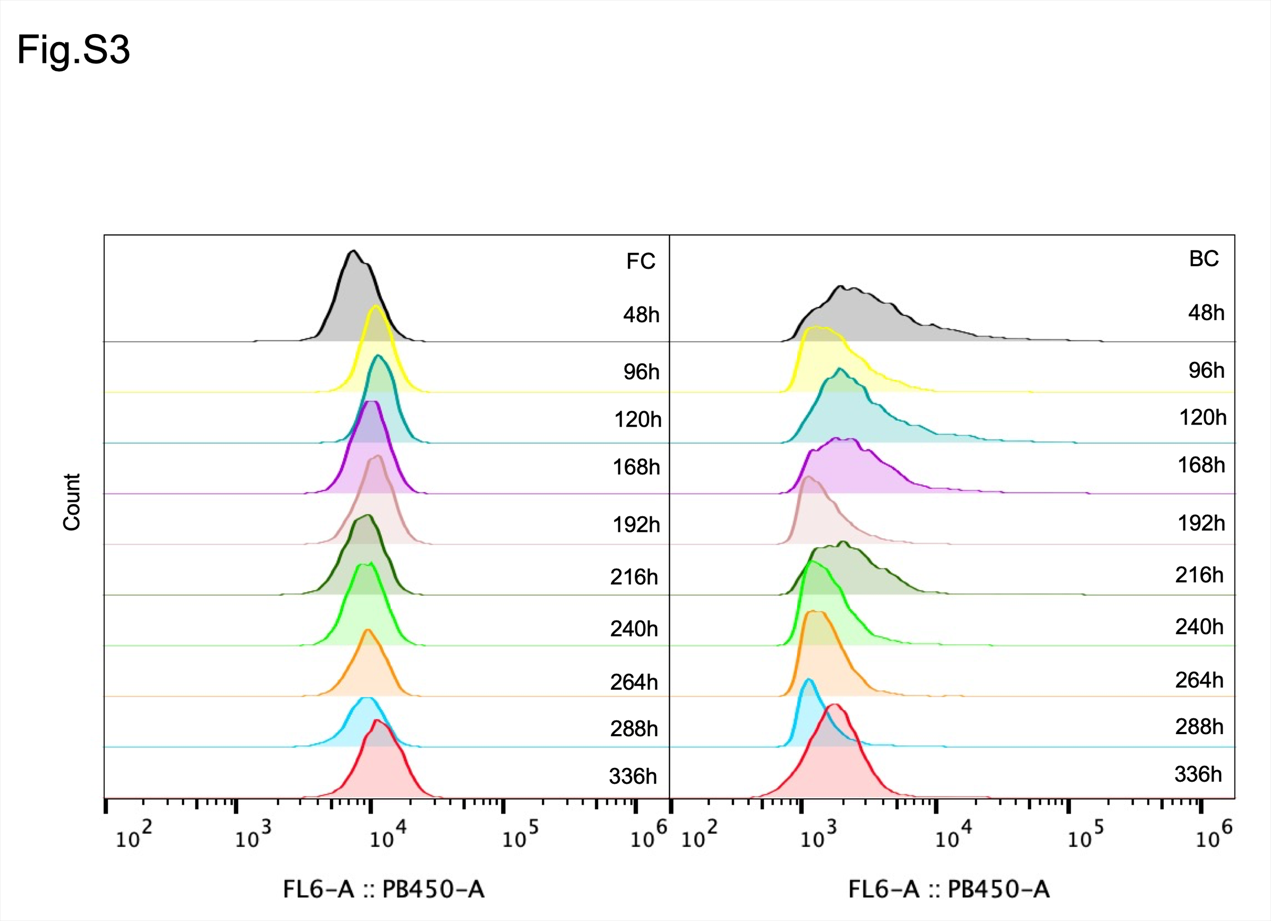
**

**Fig. S3** Comparison of DNA patterns between Cg-0206 biofilm cells (BC) and free cells (FC) during repeated-batch fermentation. Samples were collected every time at 24 h after the start of each new batch.

**Fig. S4**

**
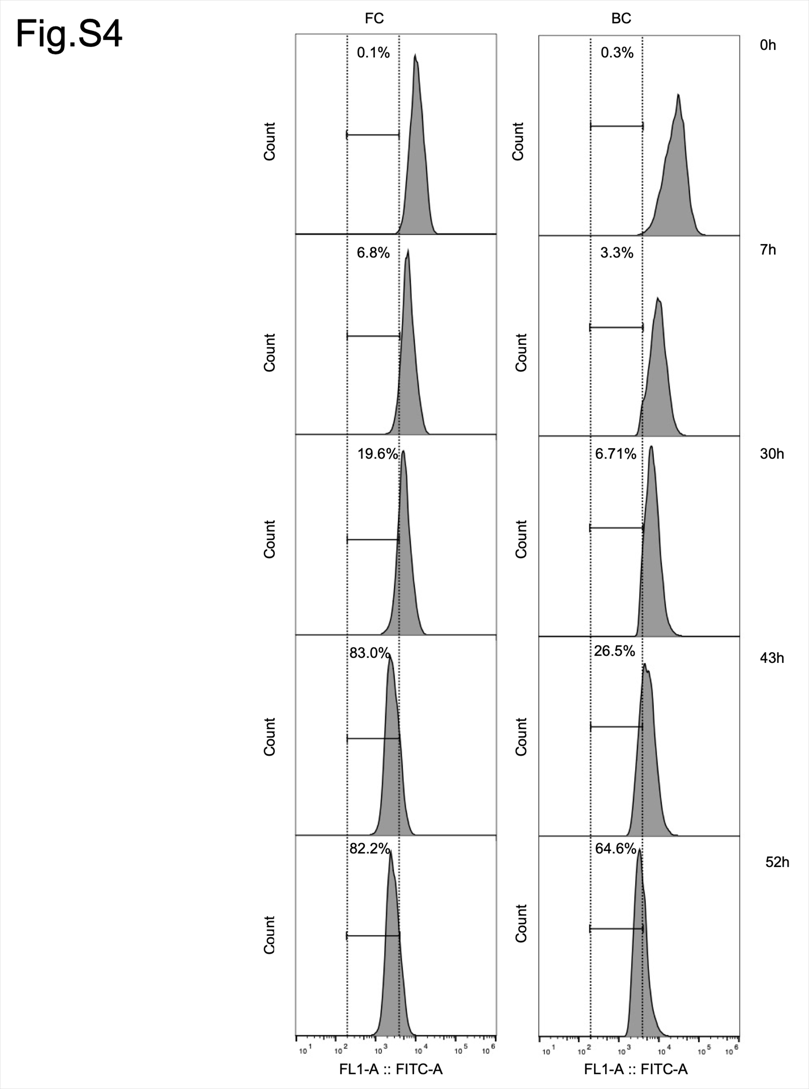
**

**Fig. S4** Cell proliferation related to Fig.5A was analyzed by CytoFLEX flow cytometer and Flow jo software (Liu et al., 2006). *C. glutamicum* ATCC13032 cells taken at the log phase of batch 2 (59 h). Cells were collected and stained with CFDA-SE, and then resuspended into fresh culture medium (0 h) for proliferation. Dilution of fluorescence (Han et al., 2009; Parish et al., 2009) was recorded at predetermined time intervals using CytoFLEX flow cytometer. The region between the two vertical dotted lines represents a control region (proliferating cell region). The numbers in these regions indicate the percentages of cells whose fluorescence intensity fell into the control region. FC, Free cells; BC, Biofilm cells.

**Fig. S5**


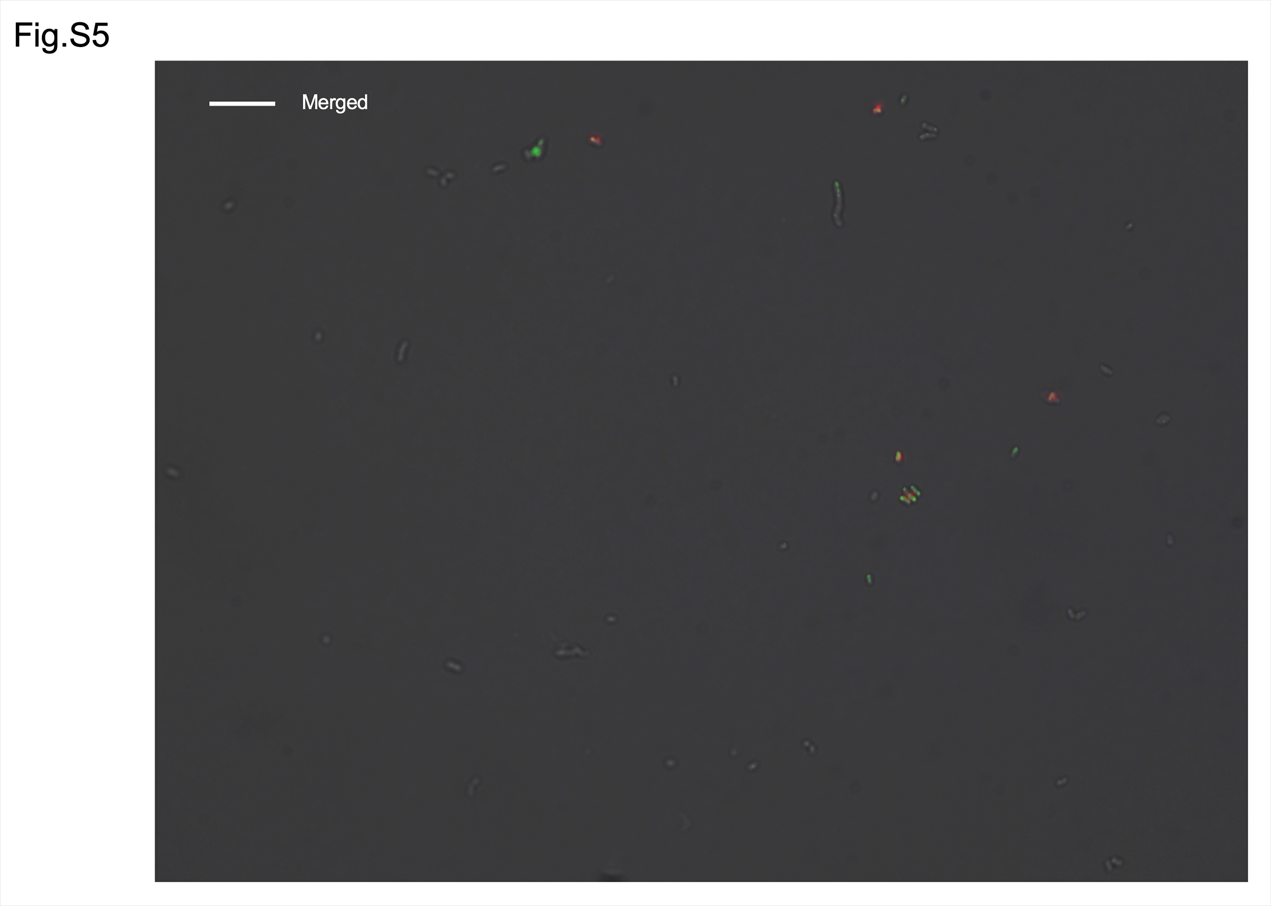


**Fig. S5** Annexin V-FITC (green) and PI (red) stained biofilm cells were visualized using an inverted fluorescence microscope MF53 (MSHOT, China). Merged images of red and green fluorescence and bright-field were shown. Scale bar, 10 µm.

**References**

Han, Y., Guo, Q., Zhang, M., Chen, Z., Cao, X. 2009. CD69+ CD4+ CD25− T cells, a new subset of regulatory T cells, suppress T cell proliferation through membrane-bound TGF-β1. *The Journal of Immunology*, **182**(1), 111-120.

Liu, D., Yu, J., Chen, H., Reichman, R., Wu, H., Jin, X. 2006. Statistical determination of threshold for cellular division in the CFSE-labeling assay. *Journal of immunological methods*, **312**(1-2), 126-136.

Łos, J.M., Golec, P., Wegrzyn, G., Wegrzyn, A., Łos, M. 2008. Simple method for plating *Escherichia coli* bacteriophages forming very small plaques or no plaques under standard conditions. *Applied and environmental microbiology*, **74**(16), 5113-5120.

Parish, C.R., Glidden, M.H., Quah, B.J., Warren, H.S. 2009. Use of the intracellular fluorescent dye CFSE to monitor lymphocyte migration and proliferation. *Current protocols in immunology*, **84**(1), 4.9. 1-4.9. 13.
